# Optical biopsy of nasal cavity cancer with confocal laser endomicroscopy – a pilot study

**DOI:** 10.1007/s00405-025-09968-8

**Published:** 2026-02-16

**Authors:** Flurin Müller-Diesing, Matti Sievert, Bharat Akhanda Panuganti, Marc Aubreville, Nils Porsche, Stephan Hackenberg, Manuel Stöth, Agmal Scherzad, Markus Wirth, Philipp Winnand, Miguel Goncalves

**Affiliations:** 1 Department of Oto-Rhino-Laryngology, Head and Neck Surgery, Wurzburg University Hospital, Wurzburg, Germany; 2 Department of Otorhinolaryngology, Head and Neck Surgery, Friedrich-Alexander-Universität Erlangen-Nürnberg, University Hospital, Erlangen, Germany; 3 Department of Otolaryngology- Head and Neck Surgery, Washington University in St. Louis, St. Louis, MO USA; 4 Flensburg University of Applied Sciences, Flensburg, Germany; 5 Department of Otorhinolaryngology, Head and Neck Surgery, Uniklinik RWTH Aachen University, Aachen, Germany; 6 Department of Oral and Maxillofacial Surgery, Uniklinik RWTH Aachen University, Aachen, Germany

**Keywords:** Confocal laser endomicroscopy, Nasal cavity carcinoma, Optical biopsy

## Abstract

**Purpose:**

Nasal cavity carcinoma (NCC) arises in an anatomically and aesthetically critical region. Complete resection with negative margins is mandatory, yet even minimal additional tissue removal may cause substantial cosmetic and functional morbidity and increase the need for reconstruction. This underscores the need for an intraoperative optical biopsy. We report the first evaluation of confocal laser endomicroscopy (CLE) as a potential tool for optical biopsy in NCC.

**Methods:**

In this pilot feasibility study including six patients, a blinded evaluation of 68 CLE video sequences (34 normal mucosa, 34 tumor), containing 4500 images, was performed independently by three investigators. A previously established scoring system from CLE studies in head and neck squamous cell carcinoma was applied to determine NCC-specific cut-off values and to assess concordance with investigator-based evaluations.

**Results:**

Compared with normal mucosa, tumor sequences exhibited greater structural heterogeneity, atypical vascular patterns, increased variation in cell size, and indistinct cell borders. Investigators achieved an average sensitivity of 86.27% ± 9.1 and specificity of 94.13% ± 6.36. The comparison between all expert-based evaluations and all score-based results demonstrated an overall substantial agreement (Fleiss’ kappa: 0.75).

**Conclusion:**

CLE appears feasible as an intraoperative optical biopsy in nasal cavity carcinoma. Prospective clinical studies should test clinical utility such as rates of complete resection, defect size, margin sampling efficiency.

## Introduction

Squamous cell carcinoma of the nasal cavity is a rare malignancy. Due to its low incidence, it is often grouped with squamous cell carcinoma of the paranasal sinuses, together accounting for 0.35/100,000 cases per year in Europe [1]. Consequently, data on the treatment of nasal cavity carcinoma (NCC) are limited compared to other head and neck tumors. The 5-year survival rate of 49.5% for sinonasal carcinomas underscores the need to optimize therapy [1].

Surgical resection remains the standard treatment, often followed by adjuvant radiotherapy or chemoradiotherapy to improve survival [2–4]. Achieving negative resection margins is critical, as local recurrence is the most common pattern of treatment failure [3–5]. Robin et al. emphasize that tumors with a better surgical accessibility, such as NCC, are generally associated with a more favorable prognosis compared to less accessible sinonasal carcinomas [3].

However, surgical accessibility comes with aesthetic challenges. The nose is a very vulnerable structure from an aesthetic point of view, and in many cases, preservation is impossible, requiring partial or total rhinectomy [2]. Even with optimal postoperative epithesis care, significant psychological impacts are observed [6]. Thus, the primary challenge is to achieve oncologically safe margins while preserving as much of the aesthetic nasal structure as possible.

Histopathologic frozen section analysis is the current intraoperative standard to achieve this goal. While reliable, it is time-consuming, and the surgeon receives feedback only after tissue resection [7].

These limitations highlight the potential of an optical in vivo biopsy. Confocal laser endomicroscopy (CLE) provides real-time, high-resolution imaging of superficial tissue layers by activating a fluorescent dye, which is detected simultaneously by the CLE probe. Focal depth varies with the probe (40–70 µm or 55–65 µm), with a field of view of 240 µm or 325 µm [8].

CLE has already been successfully applied to numerous oncological questions across various medical specialties [9]. It is widely used in gastroenterology to evaluate esophageal adenocarcinoma, Barrett’s esophagus, and cystic pancreatic lesions [10], and in pulmonology and urology to detect lung and urothelial carcinomas [11, 12]. In the head and neck, CLE has shown high sensitivity for detecting oral cavity, laryngeal, and oropharyngeal carcinomas (90%) [1, 13], with excellent interrater reliability [13].

Building on these results, we hypothesized that CLE can reliably differentiate NCC from adjacent non-neoplastic mucosa. To our knowledge no structured studies have addressed the use of CLE in this tumor entity. Given the anatomical complexity and aesthetic significance of the nasal region, a real-time, non-invasive diagnostic tool would be particularly valuable. Therefore, this study aims to evaluate the feasibility of CLE in the nasal cavity, focusing on its potential to assist in the delineation of tumor margins.

## Materials and methods

**Fig. 1:**
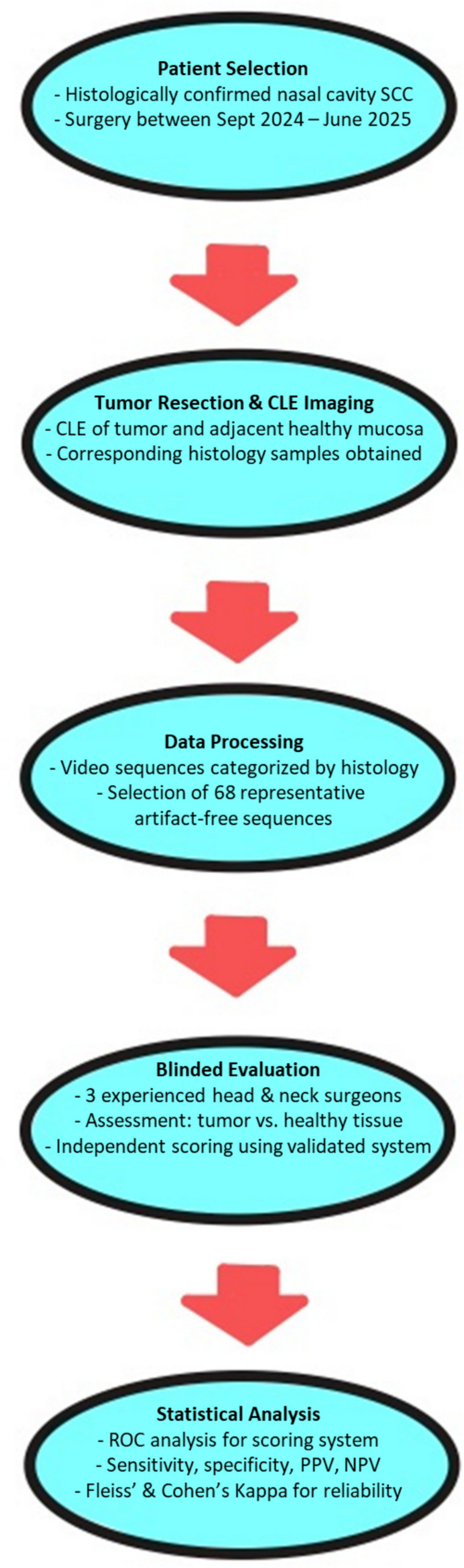
Flow diagram of the study process

In the present study, we conducted a blinded evaluation of CLE video sequences of healthy tissue as well as NCC by three different investigators. Each individual sequence was correlated with a corresponding histopathological reference. Based on the results of the blinded evaluation, sensitivity, specificity, positive predictive value, and negative predictive value were calculated. Furthermore, we are evaluating a scoring system, previously established in studies on other head and neck squamous cell carcinomas (HNSCC), for its applicability in NCC [13] (Fig. 1).

### Tumor resection and CLE examination

The CLE sequences were obtained from a cohort of patients who underwent surgery at our center between September 2024 and June 2025. All patients had a histologically confirmed squamous cell carcinoma of the nasal cavity prior to surgery. Surgical resection was performed under general anesthesia. Immediately prior to resection, CLE imaging was conducted to assess the resection margins. For this purpose, 2.5 ml of fluorescein (Fluorescein Alcon 10%, Alcon PHARMA GmbH, Freiburg, Germany) was administered intravenously (optionally, a second dose of 2.5 ml fluorescein was given to prolong the examination time when necessary). Subsequently, examination was performed using the CLE probe (GastroFlex probe with Cellvizio laser system, Mauna Kea Technologies, Paris, France). Images were acquired both in the area of the macroscopically visible tumor and in the surrounding macroscopically healthy tissue. In this process, the macroscopically healthy tissue was biopsied with a safety margin of at least 10 mm from the macroscopically visible tumor boundary. The video sequences were labeled and stored separately. The corresponding tissue areas in the surgical field were marked and sent separately for histopathological analysis. This approach enabled us to obtain corresponding histological results for each recorded video sequence.

### Processing and analysis of CLE sequences

CLE video sequences, categorized according to their corresponding histology, were reviewed by the first author using Cellvizio Viewer software 1.6.2 (Mauna Kea Technologies, Paris, France). From the raw data video sections were selected according to the following criteria:

1. clear visualization of cells and/or vessels,
2. absence of motion artifacts that could obscure or distort the image,
3. no probe contamination compromising image quality (e.g., due to blood)
4. exclusion of redundant recordings from the same anatomical region.

A total of 68 (34 from healthy tissue and 34 from NCC) representative, artifact-free sequences were selected, randomized manually by the first author, and presented to the three investigators (senior authors) in a blinded fashion.

The investigators, all head and neck surgeons with at least five years of CLE experience, based their assessments on prior experience with CLE imaging of other HNSCC but, in the absence of previous systematic investigations, without specific knowledge of CLE in the nasal cavity.

Each sequence was evaluated in two ways. First, the investigators judged whether it represented NCC or healthy tissue, relying solely on their experience. Independently, they completed a previously validated scoring system (Table 1), previously applied by the research group in studies of other HNSCC [13]. The scoring was not intended to allow the investigators themselves to distinguish between tumor and healthy tissue. Instead, the scoring results were subsequently analyzed by the first author. A ROC analysis was performed to determine the optimal cut-off value, which was then used to calculate sensitivity and specificity. These metrics were compared with the investigators’ experience-based assessments.

**Table 1:** Score used in the evaluation to distinguish between squamous cell carcinoma and healthy tissue, taken from previous work by the research group on HNSCC of the oral cavity, larynx, and pharynx [12, 14, 15]

	**Tissue homogeneity**	**Cell size**	**Cell cluster**	**Blood vessels**	**Nucleus/cytoplasma ratio**	**Cell borders**
Criterion of non-neoplastic mucosa	Homogeneous: 0	Uniform: 0	Honeycomb: −1	Capillary loops: −1	No small dark cells: 0	Clear cell borders: 0
Criterion of malignancy	Inhomogeneous: 1	Different: 1	No honeycomb: 1	Atypical vessels: 0	Small/dark cells: 1	Disrupted cell borders: 1

### Statistical analysis

All data were transferred to standard spreadsheets for analysis. Statistical analyses were performed using SPSS Statistics version 26.0 (IBM, Armonk, NY, USA). Graphical analysis was performed using GraphPad Prism (Graphpad Software Inc., San Diego, USA). Sensitivity, specificity, positive predictive value, and negative predictive value were calculated for each examiner. The interrater reliability among the three different investigators was assessed using Fleiss’ Kappa. The interrater reliability between each investigator’s assessment and their respective scoring results was evaluated using Cohen’s Kappa. Unless otherwise specified, all data are presented as mean ± standard deviation. A p-value of less than 0.05 was considered statistically significant.

CLE examinations and surgical procedures were conducted following written informed consent from all participants. This study received approval from the Institutional Ethics Committee for Human Research at Julius-Maximilians-University Würzburg (reference number: 154/23_mpz-sc).

## Results

### Demographics

During the observation period, we included data from a total of six different patients. The tumors were located throughout the nasal cavity, and cases across all histopathological grading categories were represented (see Table 2). In total, 68 CLE sequences (34 tumor/34 healthy) comprising 4,500 images were analyzed.

**Table 2:** Tumor localization and TNM classification. Tumors from all regions of the nasal cavity are represented

Patient	Sex	TNM	Tumorsite
1	M	pT4a pN0 M0 G2	involving the entire left nasal cavity, origin not identifiable
2	F	pT2 cN0 M0 G1	lateral nasal wall
3	M	pT2 pN0 M0 G3	lateral nasal wall
4	M	pT1 pN0 M0 G2	left septum
5	M	pT2 pN0 M0 G3	left middle turbinate
6	M	pT1 pN0 M0 G3	right septum

### Analysis independent of the score

First, we present the analysis without a scoring system, based on the investigators’ experience in evaluating CLE sequences from various other tumor entities. The sensitivities of the three investigators were 73.5%, 91.2%, and 94.1%, while the specificities were 85.3%, 97.1%, and 100%. On average, a sensitivity of 86.27% ± 9.1 and a specificity of 94.13% ± 6.36 were obtained (see Table 3).

**Table 3:** Results of the investigators’ analysis without a scoring system in % (95%-CI)

	**Examiner 1**	**Examiner 2**	**Examiner 3**	**Average**
Accuracy	79.4	94.1	97.1	90.2 ± 7.73
Sensitivity	73.5 (55.6% – 87.1%)	91.2 (76.4% – 98.1%)	94.1 (81.3% – 99.3%)	86.27 ± 9.1
Specificity	85.3 (68.9% – 94.0%)	97.1 (85.1% – 99.9%)	100 (88.8% – 100%)	94.13 ± 6.36
Positive predictive value	83.3	96.9	100	93.4 ± 7.25
Negative predictive value	76.3	91.7	94.4	87.47 ± 7.98

Regarding inter-rater reliability, a Fleiss’ kappa of 0.68 was obtained. According to the Landis and Koch scale, this corresponds to substantial agreement.

In summary, based on their prior experience in CLE evaluation, all investigators were able to reliably differentiate between tumor and healthy tissue. A substantial level of interobserver agreement was observed.

### Analysis based on the scoring system

**Fig. 2:**
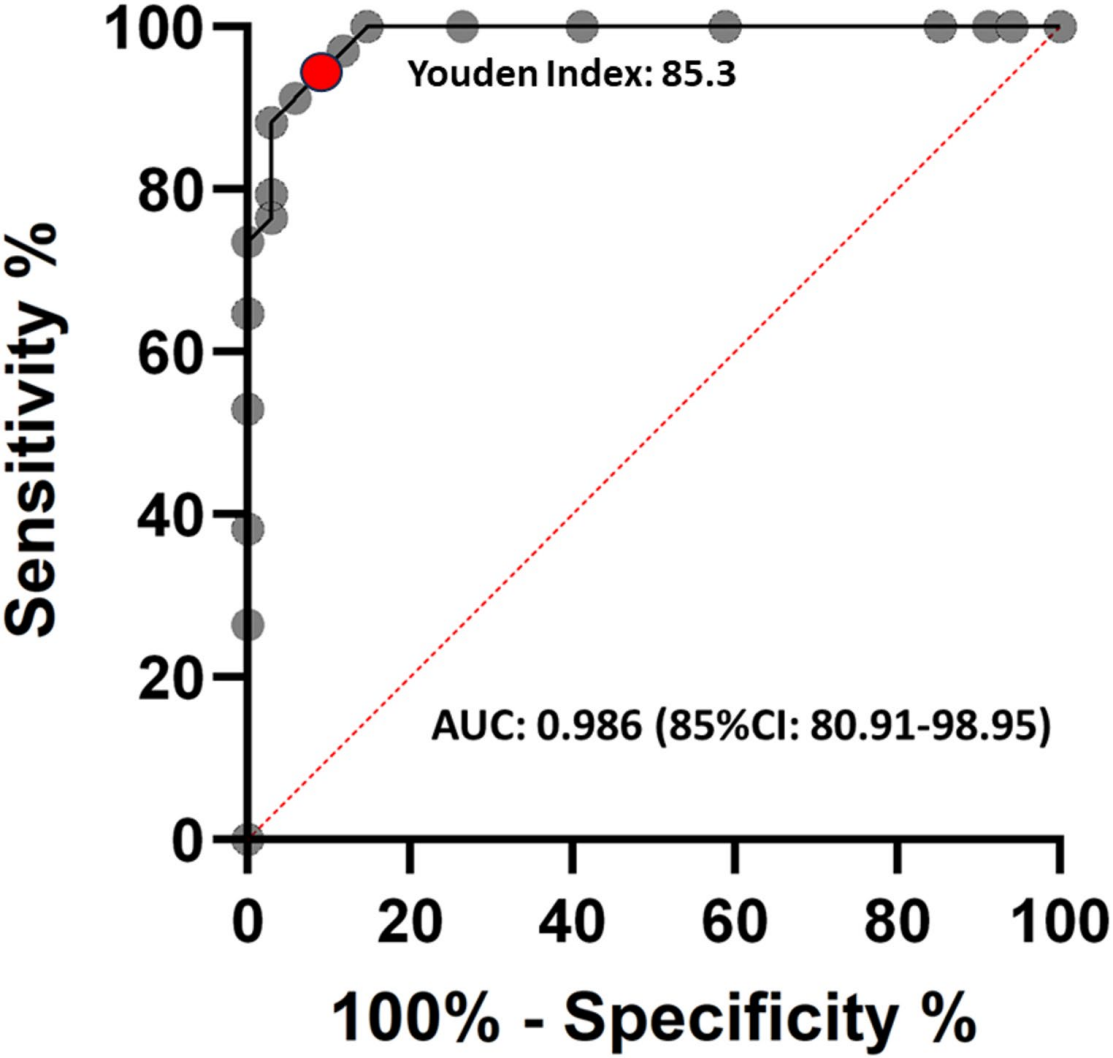
ROC curve based on the mean values of the investigators’ scoring results

All sequences were additionally assessed using the predefined scoring system. The first author analyzed the scoring results, and a ROC analysis identified the highest Youden index (85.3) for a scoring value of ≥1, representing the threshold that best discriminates between tumor and healthy tissue (Fig. 2). Applying this cut-off retrospectively to the investigators’ scores yielded sensitivities of 88.2%, 94.1%, and 100%, and specificities of 55,9%, 97.1%, and 94.1%, with mean sensitivity and specificity of 94.1% ± 4.82 and 82.37% ± 18.75, respectively. The individual AUC values of the investigators were 0.865, 0.973 and 0.995. All p-values for the examiners were <0.0001.

The agreement between each investigators’ experience-based assessment and the scoring-based evaluation was calculated. The score and the examiners’ assessments disagreed in 14, 4, and 1 cases, respectively. For the three evaluators, Cohen’s kappa values of 0.52, 0.85, and 0.88 were obtained, corresponding to moderate agreement in the first case and almost perfect agreement in the other two cases, according to Landis and Koch.

Furthermore, the overall interrater agreement between all experience-based and all score-based evaluations was assessed. The analysis yielded a Fleiss’ kappa of 0.75, indicating a substantial level of agreement according to the classification by Landis and Koch.

### Characteristics of NCC CLE sequences

**Fig. 3:**
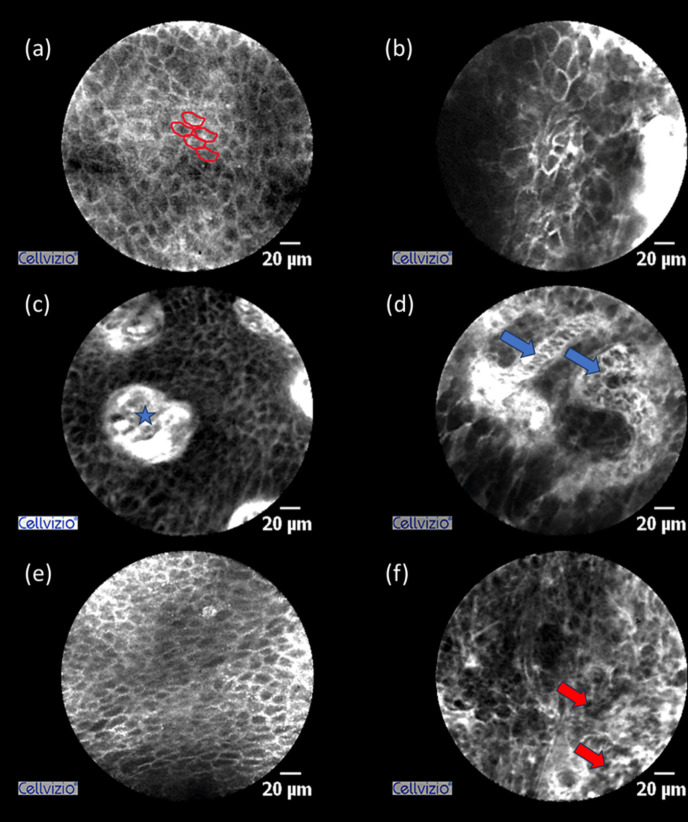
Single frames from the video sequences. On the left side (a,c,e) showing healthy mucosa, with (a) highlighting the homogeneous, small cells arranged in the typical honeycomb-like pattern (marked in red), (c) depicting a characteristic, sharply demarcated round vessel (marked with a blue asterisk), and (e) showing again the typical honeycomb-like pattern, with uniformly shaped cells. On the right side (b, d, f), NCC is shown, in (b) with inhomogeneous tissue architecture and cells in different sizes with disrupted cellborders, in (d) blue arrows are indicating an irregularly shaped vessel and red arrows in (f) are highlighting small dark cells as a typical tumor feature

Finally, the typical features of CLE sequences from NCC are summarized. Tumor sequences demonstrated a markedly heterogeneous tissue architecture, with cells of variable size and often indistinct margins. On average, tumor cells appeared noticeably larger than those in healthy tissue. In some areas, individual cells could no longer be delineated. Tumor vessels exhibited irregular margins and heterogeneous flow. By contrast, healthy tissue showed uniform, sharply defined cells arranged in a honeycomb-like pattern, and vessels with clearly defined margins and a predominantly round to oval shape (Fig. 3).

## Discussion

To the best of our knowledge, there has been no systematic evaluation of CLE in squamous cell carcinoma of the nasal cavity to date. Current literature provides only very limited data on the application of CLE for the detection of paranasal sinus malignancies. One report describes two cases of malignant melanoma in which CLE was successfully employed [14], while another study investigating various paranasal sinus pathologies included a single case of adenocarcinoma but no squamous cell carcinoma [16]. Additionally, there is a report of a single case of paranasal sinus squamous cell carcinoma examined with CLE [17].

Consistent with that single case report, the present study demonstrates similar tissue characteristics of squamous cell carcinoma of the nasal cavity as visualized with CLE. In both the study by Neda et al. and in our investigation, CLE images revealed a heterogeneous tissue architecture, irregular cellular morphology, variations in the nuclear-to-cytoplasmic ratio, and abnormal capillary structures [17].

Given the absence of systematic evaluation to date, the sensitivity and specificity observed in this study should be interpreted in the context of prior CLE investigations of HNSCC at other anatomical sites. A similarly designed evaluation of CLE images for the detection of oropharyngeal carcinomas yielded a sensitivity of 90% and a specificity of 79% [1]. Another study differentiating CLE sequences of laryngeal squamous cell carcinomas from healthy laryngeal mucosa based on tissue homogeneity reported a sensitivity of 81.8% and a specificity of 86.2% [18]. Against this background, our feasibility study produced promising results, with an average sensitivity of 86.27% and specificity of 94.13% for the detection of squamous cell carcinoma of the nasal cavity using CLE.

A limitation of the current analysis is that investigators’ assessments were subjective and based on prior experience with other tumors. Optimizing CLE application requires an objective evaluation framework. To this end, we applied a scoring system previously established for HNSCC at other anatomical sites [13]. In prior studies employing this system, sensitivities of 95.1% and 90.4% and specificities of 87.4% and 86.4% were achieved [13]. Although the scoring system was not primarily used for sequence evaluation in our study, a retrospective analysis after defining the ROC-derived cut-off value yielded an average sensitivity of 94.1% and specificity of 82.37%. Comparison of scoring-based evaluation with expert assessments showed moderate agreement in one case and almost perfect agreement in two cases. These findings indicate that the scoring system is suitable for application in squamous cell carcinoma of the nasal cavity. Previous investigations of the score in other HNSCC demonstrated that even inexperienced investigators could achieve reliable results [13]. Further studies assessing the use of the score by inexperienced investigators in squamous cell carcinoma of the nasal cavity would represent an important step toward implementing CLE in clinical practice.

A further limitation of our study is that a single investigator selected the blinded sequences. While redundant videos were removed to prevent the examiners from evaluating the same tissue twice, some selection bias cannot be fully excluded.

An important strategy to address both the limitations of the study design and to further objectify data analysis is the use of computational tools or artificial intelligence (AI). On one hand, a trained AI could assist in selecting representative, artifact-free sequences, thereby reducing selection bias. On the other hand, AI represents a promising approach for the analysis of CLE sequences. If AI is able to reliably distinguish between healthy tissue and tumor, CLE assessment could become largely independent of the investigator. In this regard, previous work on other tumor entities has already shown promising results [9].

For successful clinical implementation of CLE, an objective evaluation framework must be accompanied by a clear definition of its intended clinical application. When assessing resection margins, comparison with the current gold standard—frozen section analysis—is particularly relevant. The average sensitivity of CLE in our study was comparable to reported frozen section sensitivity (approximately 93.8%) [7]. A clear advantage of CLE over frozen section is the immediate in vivo visualization, allowing the investigator to receive direct feedback and requiring significantly less time. In contrast, CLE is associated with high costs [19]. The limited penetration depth of CLE (40–70 µm) also represents a limitation [8]. Tumor extension in depth can only be assessed to a limited extent using CLE. However, CLE should not be viewed as a competitor to frozen section, but could rather be a complementary tool. CLE provides immediate, real-time feedback to the surgeon, while frozen section analysis can still be performed in parallel on margin biopsies.

Determining the optimal strategy—CLE alone, frozen section alone, or a combination—will require additional comparative studies. Moreover, further research is needed to address the limitations of the present work. As a feasibility study, our results represent an initial step that should be followed by investigations with larger patient cohorts and a standardized evaluation protocol.

This pilot study demonstrates two key points: first, that CLE can be successfully applied by experienced investigators in squamous cell carcinoma of the nasal cavity, and second, that a previously established HNSCC-derived scoring system is generally applicable in this context. These findings address feasibility and diagnostic performance, but patient-relevant outcomes were not assessed. Generalizability is limited by the small, single-center cohort, curated image clips, and evaluation by experienced investigators. Successful clinical translation will require standardized image acquisition, predefined decision thresholds, and integration of CLE into a margin-assessment workflow alongside frozen section. CLE represents a promising complementary intraoperative tool for margin assessment, and future multicenter studies incorporating AI-based image analysis are warranted to validate diagnostic thresholds and workflow integration. Ultimately, demonstration of clinical utility—such as higher R0 resection rates, smaller defects, and more efficient margin sampling—will require prospective intraoperative trials with appropriate endpoints and timing metrics.

## Data Availability

All data are available on request from the corresponding author.
